# Upadacitinib for refractory cutaneous disease in a patient with tif1γ-positive dermatomyositis^؆^

**DOI:** 10.1016/j.abd.2026.501443

**Published:** 2026-09-04

**Authors:** Yusuf Demir, Hande Ogun, Bercemhan Sulu, Muazzez Cigdem Oba, Emire Seyahi

**Affiliations:** a Department of Dermatology and Venereology, Faculty of Medicine, Istanbul University-Cerrahpaşa, Istanbul, Turkey; b Department of Internal Medicine, Division of Rheumatology, Faculty of Medicine, Istanbul University-Cerrahpaşa, Istanbul, Turkey

Dear Editor,

A 49-year-old woman presented facial and truncal erythema, progressive proximal weakness, and dysphagia. Physical examination showed typical signs of classic dermatomyositis, including heliotrope rash, Gottron papules over the metacarpophalangeal and interphalangeal joints, and V-sign erythema (Fig. 1). Serum creatine kinase level was elevated at 730 U/L (<90 U/L). The myositis-specific antibody panel was positive for anti-Transcription Intermediary Factor 1-gamma (TIF1γ) antibody. Given the malignancy risk associated with TIF1γ positivity, cancer screening was performed according to the International Guideline for Idiopathic Inflammatory Myopathy-Associated Cancer Screening.^2^ This included a gynecological examination, serum tumor markers, mammography, colonoscopy, cross-sectional Computed Tomography (CT) imaging of the neck, thorax, abdomen, and pelvis, and Positron Emission Tomography-CT, which were all negative. Baseline assessment showed a Cutaneous Dermatomyositis Disease Area and Severity Index (CDASI) activity score of 26, consistent with moderate-to-severe cutaneous activity, and muscle weakness with Manual Muscle Testing-8 (MMT-8) score of 64/80.

**Fig. 1:**
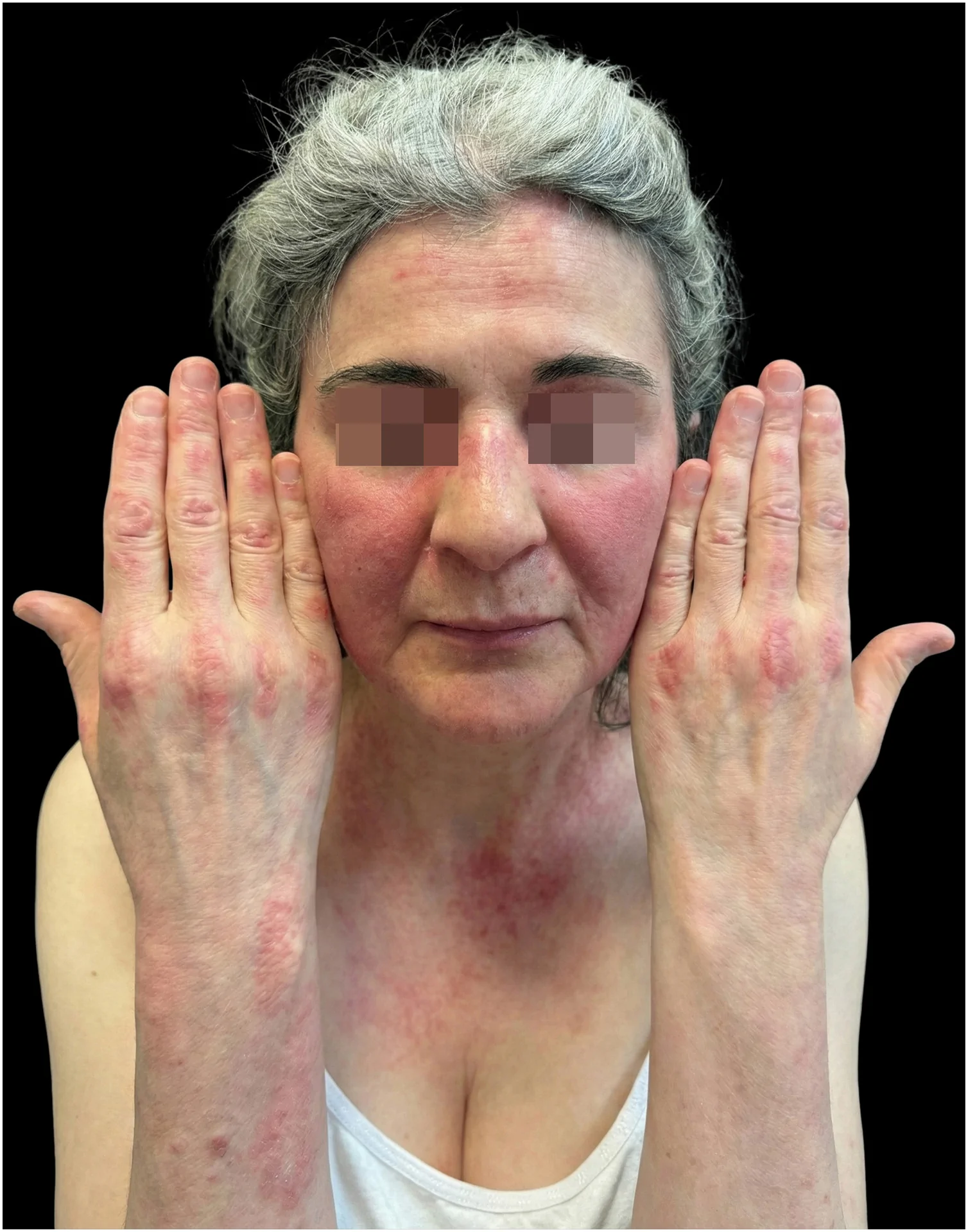
Clinical presentation at baseline. Heliotrope rash with periorbital erythema, V-sign erythema on the anterior chest, and Gottron papules over the metacarpophalangeal and interphalangeal joints. CDASI activity score: 26.

Treatment with prednisolone (1 mg/kg/day) and mycophenolate mofetil (2 g/day) was started. After 4-months, rituximab was added due to inadequate response in both cutaneous and muscular domains. However, two courses of rituximab failed to achieve remission. Intravenous immunoglobulin was inaccessible due to reimbursement limitations, and corticosteroid dosing regimens were limited by the compression fracture.

Three months after the last rituximab infusion, upadacitinib, a selective Janus Kinase 1 (JAK1) inhibitor, was initiated at 15 mg daily, with prednisolone maintained at 5 mg/day and mycophenolate mofetil continued at 2 g/day. At 4-months, muscle strength improved with normalized CK, increased MMT-8 scores (78/80), and resolved dysphagia. However, due to persistent active cutaneous disease (CDASI activity score 20), the dose was increased to 30 mg daily. By week 4, a major clinical response as defined by ACR/EULAR (American College of Rheumatology/European League Against Rheumatism) criteria was achieved, with marked improvement in cutaneous disease activity (CDASI activity score 6) (Fig. 2). At month-12, the patient remains in remission, and 12-month malignancy surveillance tests are negative.

**Fig. 2:**
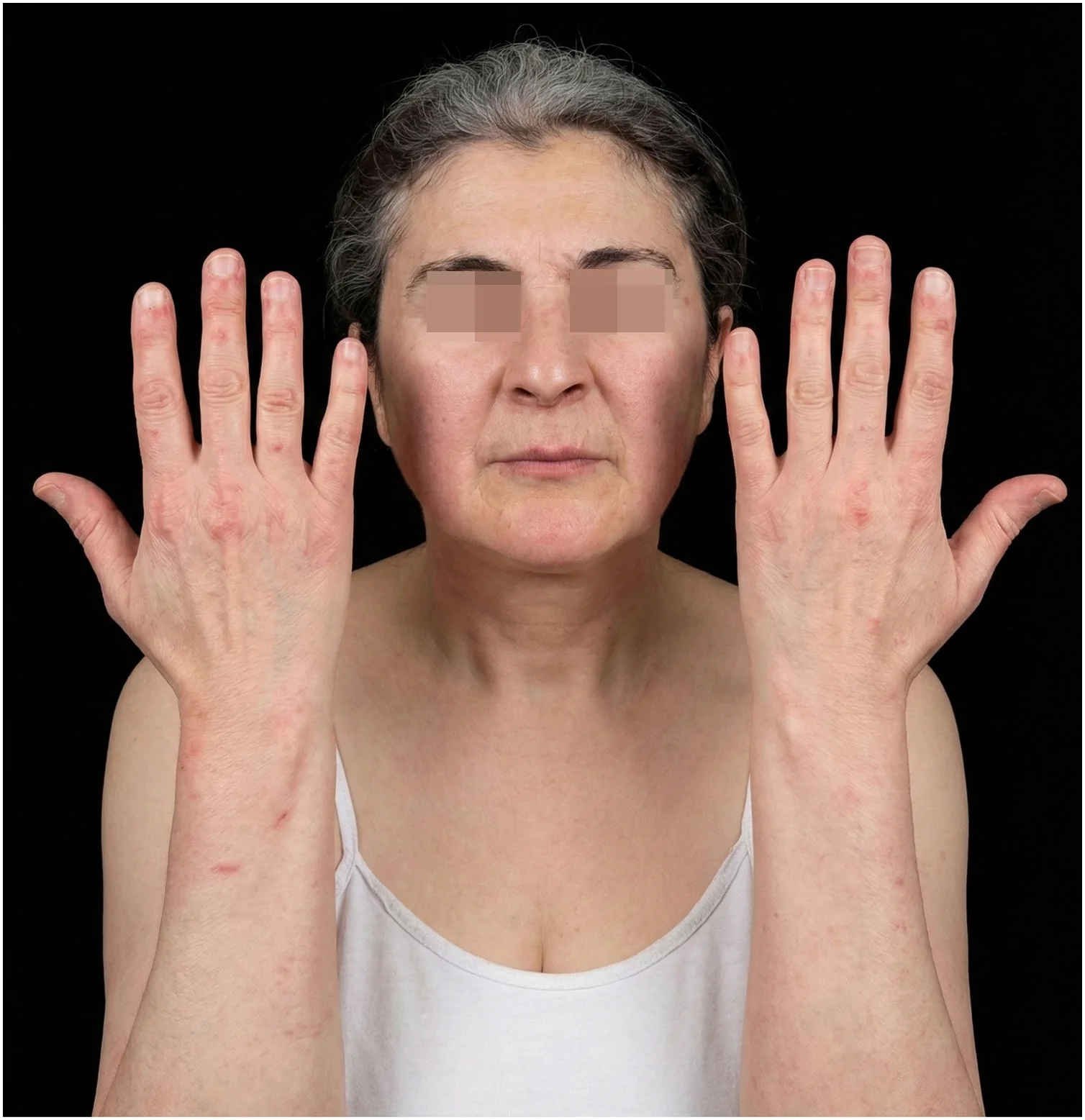
Clinical response 4-weeks after upadacitinib dose escalation to 30 mg daily. Near-complete resolution of facial erythema and V-sign, with significant fading of Gottron papules. CDASI activity score: 6.

The pathogenesis of dermatomyositis involves dysregulation of type I interferon signaling mediated through JAK1 and Tyrosine Kinase-2 (TYK2).^3^ JAK inhibitors, most commonly tofacitinib, are increasingly used off-label in refractory cases; upadacitinib, with its greater JAK1 selectivity, has more recently demonstrated efficacy even in patients refractory to other JAK inhibitors, underscoring the dominance of the JAK1 pathway.^4^, ^5^, ^6^, ^7^

Although existing case series and systematic reviews have established the general efficacy of JAK inhibitors in refractory dermatomyositis,^4^, ^5^, ^6^ organ-specific differential responses at distinct upadacitinib dose levels have not, to our knowledge, been previously described (Table 1).^2^, ^4^, ^5^, ^8^ In this case, muscular disease responded at 15 mg/day, whereas cutaneous disease required escalation to 30 mg/day ‒ a pattern suggesting a higher threshold of JAK1 inhibition in the skin than in muscle, consistent with the established correlation between cutaneous disease activity and type I interferon burden in the cutaneous compartment.^3^ The most comparable published case ‒ a TIF1γ-positive patient reported by Azari et al.^8^ ‒ was initiated directly at 30 mg/day, precluding any assessment of organ-specific dose-response. Furthermore, despite a prior breast cancer history, no structured malignancy surveillance was reported in that case.

**Table 1:** Comparison of the present case with published anti-TIF1γ-positive dermatomyositis cases treated with upadacitinib.^2^, ^4^, ^5^, ^8^

Feature	Present case	Azari et al. 2025^8^	Beckett et al. 2024, Patient 4^4^	Zhou et al. 2024^5^
DM subtype	Classic DM	Classic DM	Classic DM	Clinically amyopathic DM
*Patient and disease characteristics*				
Age (years)/sex	49/F	60/F	86/F	67/F
Myositis-specific antibody	Anti-TIF1γ	Anti-TIF1γ, anti-Ro52	Anti-TIF1γ	Anti-TIF1γ (ANA 1:160)
Baseline CDASI activity	26	43	50	50
Baseline MMT-8	64/80	104/150	127/150	*Normal muscle strength (amyopathic)*
Prior malignancy	None	ER/HER2-positive breast cancer (2014, in remission)	*Not reported*	None
*Prior treatment*				
Number of prior systemic agents	3 (PDN, MMF, rituximab)	5 (PDN, IVIG, MTX, rituximab, MMF)	3 (PDN, AZA, MMF)	7 (PDN, HCQ, MTX, MMF, IVIG, rituximab, tofacitinib)
Prior rituximab	Yes (2 cycles)	Yes (2 doses)	No	Yes (3 cycles)
Prior JAK inhibitor	No	No	No	Tofacitinib 5–11 mg/day (initial response with loss of efficacy at 12 months)
*Upadacitinib regimen*				
Starting dose	15 mg/day	30 mg/day	*Not specified*	15 mg/day
Dose escalation	15 → 30 mg/day (for persistent cutaneous disease)	None	*Not reported*	None
Differential organ-level response	Muscle at 15 mg; skin at 30 mg	*Not assessable (single dose)*	*Not assessable (single dose)*	*Not assessable (amyopathic)*
Concomitant systemic therapy at initiation	PDN 5 mg/day + MMF 2 g/day	*Not specified*	PDN 5 mg/day (UPA monotherapy)	HCQ 200 mg twice daily
*Outcomes*				
Cutaneous response (CDASI)	26 → 6 (4 weeks after escalation)	Rash significantly improved at 6 weeks	50 → 31 (4 months)	50 → 18 (12 months)
Muscular response (MMT-8)	64 → 78/80	104 → 130/150	127 → 131/150	*N/A (amyopathic)*
Follow-up duration	12 months	>12 months	4 months	12 months
Adverse events	None	1 minor flare (tolerated)	*None (individual P4); 1/10 series-wide discontinued (acneiform eruption, anti-MDA5 CDM)*	*None reported*
*Malignancy surveillance*				
Baseline cancer screening	Structured per IMACS guidance (Oldroyd, 2023)^2^	PET-CT (no recurrence); letrozole restarted precautionarily	*Not reported*	Negative (details not specified)
On-treatment surveillance	12-month reassessment negative	*Not addressed*	*Not addressed*	*Not addressed*

Present case: Highlighted rows indicate distinguishing features of the present case: dose escalation with differential organ-level response and structured malignancy surveillance in a classic dermatomyositis presentation. MMT-8 in the present case is reported on the 0–80 simple-sum scale; Azari et al. and Beckett et al. (Patient 4) used the IMACS 0–150 composite scale. Beckett et al. data reflect Patient 4, the only anti-TIF1γ-positive patient in that case series.

ANA, Antinuclear Antibody; AZA, Azathioprine; CDASI, Cutaneous Dermatomyositis Disease Area and Severity Index; CDM, Classic Dermatomyositis; DM, Dermatomyositis; ER, Estrogen Receptor; F, Female; HCQ, Hydroxychloroquine; HER2, Human Epidermal growth factor Receptor-2; IMACS, International Myositis Assessment and Clinical Studies; IQR, Interquartile Range; IVIG, Intravenous Immunoglobulin; JAK, Janus Kinase; MDA5, Melanoma Differentiation-Associated gene-5; MMF, Mycophenolate Mofetil; MMT-8, Manual Muscle Testing-8; MTX, Methotrexate; N/A, Not Applicable; PDN, Prednisone/Prednisolone; PET-CT, Positron Emission Tomography – Computed Tomography; SD, Standard Deviation; TIF1γ, Transcription Intermediary Factor 1-gamma; UPA, Upadacitinib.

The malignancy risk associated with JAK inhibitors merits particular consideration here, given the elevated paraneoplastic risk conferred by TIF1γ positivity.^1^, ^2^ The class-level safety signal derives primarily from the ORAL Surveillance trial of tofacitinib in rheumatoid arthritis, which prompted a regulatory warning extended to all JAK inhibitors. For upadacitinib specifically, pooled long-term data across seven indications in 8632 patients (27,164 patient-years) showed exposure-adjusted incidence rates of 0.2–0.9/100 patient-years for malignancy excluding non-melanoma skin cancer and 0–1.4/100 patient-years for non-melanoma skin cancer, with rates remaining stable over time,^9^ suggesting that risk does not accumulate disproportionately with prolonged exposure. These data provide reassurance but do not abolish the need for vigilance in TIF1γ-positive disease. Rather than contraindicating JAK inhibition in this serotype, these considerations reinforce the need for a structured oncological surveillance framework aligned with established guidelines^2^ ‒ a practice not systematically addressed in prior JAK inhibitor reports in dermatomyositis.

In refractory TIF1γ-positive dermatomyositis, upadacitinib 15 mg may control muscular disease, but escalation to 30 mg may be necessary to achieve better cutaneous response. However, given the increased risk of malignancy associated with this serotype, initiating JAK inhibitors and determining posology should be considered within a careful, personalized treatment strategy, and closer malignancy surveillance is required.

## Authors' contributions

Yusuf Demir: Approval of the final version of the manuscript; critical literature review; data collection, analysis and interpretation; manuscript critical review; study conception and planning.

Hande Ogun: Approval of the final version of the manuscript; data collection, analysis and interpretation; ıntellectual participation in propaedeutic and/or therapeutic management of studied cases.

Bercemhan Sulu: Approval of the final version of the manuscript; data collection, analysis and interpretation; ıntellectual participation in propaedeutic and/or therapeutic management of studied cases.

Muazzez Cigdem Oba: Approval of the final version of the manuscript; effective participation in research orientation; manuscript critical review; study conception and planning.

Emire Seyahi: Approval of the final version of the manuscript; effective participation in research orientation; ıntellectual participation in propaedeutic and/or therapeutic management of studied cases; study conception and planning.

## Financial support

None declared.

## Research data availability

Does not apply.

## Conflicts of interest

None declared.
